# On the Use of Dynamic Calibration to Correct Drop Counter Rain Gauge Measurements

**DOI:** 10.3390/s21186321

**Published:** 2021-09-21

**Authors:** Mattia Stagnaro, Arianna Cauteruccio, Luca G. Lanza, Pak-Wai Chan

**Affiliations:** 1 Department of Civil, Chemical and Environmental Engineering, University of Genova, 16145 Genova, Italy; mattia.stagnaro@unige.it (M.S.); arianna.cauteruccio@edu.unige.it (A.C.); 2 WMO/CIMO Lead Centre “B. Castelli” on Precipitation Intensity, 16145 Genova, Italy; 3 Hong Kong Observatory, Hong Kong, China; pwchan@hko.gov.hk

**Keywords:** drop counter, rain gauge, measurement accuracy, dynamic calibration

## Abstract

Dynamic calibration was performed in the laboratory on two catching-type drop counter rain gauges manufactured as high-sensitivity and fast response instruments by Ogawa Seiki Co. Ltd. (Japan) and the Chilbolton Rutherford Appleton Laboratory (UK). Adjustment procedures were developed to meet the recommendations of the World Meteorological Organization (WMO) for rainfall intensity measurements at the one-minute time resolution. A dynamic calibration curve was derived for each instrument to provide the drop volume variation as a function of the measured drop releasing frequency. The trueness of measurements was improved using a post-processing adjustment algorithm and made compatible with the WMO recommended maximum admissible error. The impact of dynamic calibration on the rainfall amount measured in the field at the annual and the event scale was calculated for instruments operating at two experimental sites. The rainfall climatology at the site is found to be crucial in determining the magnitude of the measurement bias, with a predominant overestimation at the low to intermediate rainfall intensity range.

## 1. Introduction

Surface precipitation measurements have been recognized as “indispensable, despite advances in several areas of remotely sensing of precipitation” [1]. Direct measurements of rainfall at the ground level serve as the reference for calibration of large-scale estimates obtained from remote sensors operating with contactless measuring principles (e.g., radars and sensors on board of satellite platforms).

However, surface precipitation measurements are affected by measurement errors themselves. Catching-type precipitation gauges are subject to environmental and quantification errors, environmental conditions at the collector (including wind-induced undercatch and wetting/evaporation losses), to their instrument mechanics and measuring principle (including systematic mechanical biases, sampling, and dynamical errors) [2]. Quantification errors reveal the ability of the gauge to sense and correctly measure the amount of rainwater collected.

The World Meteorological Organization (WMO) held dedicated intercomparison campaigns, both in the laboratory [3] and in the field [4], under documented conditions, to test the performance of various catching-type precipitation gauges using different measuring principles. According to the recent intercomparison of Rainfall Intensity (RI) gauges [5,6], precipitation rates were underestimated up to 15% due to quantification errorsand even higher during intense and extreme precipitation events. In a recent study, [7] found that environmental factors (wind) accounted for 10 to 23% of the underestimation in precipitation within a lowland and upland site, respectively.

The WMO recommendations [2] and new measurement quality standards issued at national [8,9] and European [10] levels require proven instrumental accuracy and reliability. To fulfil the required performance, proper laboratory calibration procedures were developed for catching-type precipitation gauges to quantify their measurement biases [4,11,12,13,14,15].

One example of a catching-type precipitation gauge is the Drop Counter (DC) rain gauge. The DC rain gauge collects rainfall in a funnel and conveys the rainwater towards a calibrated nozzle. Water is then dispensed as drops to a sensor that counts using optical or acoustic principles [16,17,18].

Due to the small volume of the dispensed drops, these instruments are characterized by a very high sensitivity, suitable to detect very light precipitation events, which is two orders of magnitude lower than for typical tipping-bucket rain gauges. The time needed by the drops to fully develop and detach is very short even during low precipitation events, e.g., approximately three seconds for an instrument with a sensitivity of 0.004 mm at RI = 5 mm h^−1^. This allows the instrument to rapidly sense any variation of the rainfall rate during precipitation events.

DC gauges were recently employed as the reference instruments for in-field rainfall measurement campaigns, to assess the performance of the more common tipping-bucket rain gauges (TBRs) [19,20]. In both works, the TBRs were previously calibrated and dynamic calibration curves were used to correct their mechanical error. Measurements provided by the three corrected TBRs showed a general underestimation with respect to the DC gauge. Radar rainfall estimates were also compared with measurements provided by a dense network of DC rain gauges by [21,22]. Recently, Benoit et al. [23] used data from a network of DC rain gauges to validate the results of a stochastic rainfall model developed to simulate high resolution (sub-kilometer) rainfall fields.

However, these instruments are not exempt from systematic measurement biases. A recent experimental study was conducted in the field by Chan et al. [24], at the weather station of the Hong Kong International Airport, to compare rainfall measurements from three co-located DC rain gauges. The authors highlighted differences in the measurements from the three gauges and attributed part of these deviations to the rain drop formation mechanism, although they do not further discuss this problem. The calibration of eight DC rain gauges was performed by Benoit et al. [23] by using a constant drop volume approach, for RI up to 20 mm h^−1^, but no correction was proposed.

In the present work, two different DC rain gauge models were tested in the laboratory using dynamic calibration to evaluate their performance at various rainfall intensities. An innovative derivation of the calibration curves based on the actual drop volume generated by the gauge nozzle is proposed rather than using the constant drop volume approach provided by manufacturer. Suitable calibration curves were derived for both instruments to account for the drop volume variation as a function of the drop dispensing frequency. Corrections were applied in post-processing to calculate the RI values and to evaluate the improved performance obtained with the correction. Moreover, the operational limitations of the two DC gauge models were investigated, and the threshold RI value for the validity of the involved measuring principle identified.

Calibration curves were then applied to 20 years of field data available from the Chilbolton Facility for Atmospheric and Radio Research (CFARR) in the UK and 2 years of data from the Hong Kong International Airport (HKIA) field test site. This allowed to quantify the impact of the constant drop volume assumption vs. full dynamic calibration on the measurement of the total precipitation amount at the annual scale.

## 2. Materials and Method

### 2.1. Model Specifications

**Figure 1:**
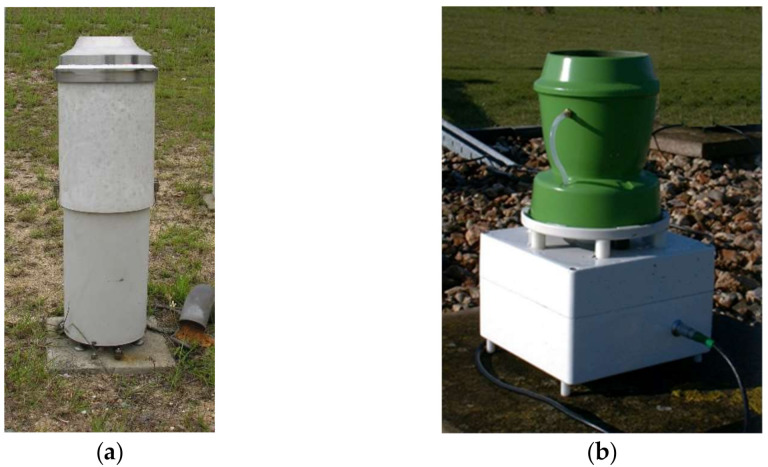
The Drop Counter rain gauges are manufactured by the Ogawa Seiki Co. Ltd. (OSC) (**a**) and the Chilbolton RAL (CRAL) (**b**).

In this study, two DC rain gauges manufactured by Ogawa Seiki Co. Ltd. (Tokyo, Japan) and the Chilbolton Rutherford Appleton Laboratory (Didcot, UK) are considered (see Figure 1), named in the following OSC and CRAL, respectively. These gauges are equipped with an optical sensor that detects the dispensed drops, allowing to count the number of drops within a given period. The drop frequency is related to the rainfall intensity, and usually total rainfall is calculated by assuming a constant volume of the drops dispensed by the nozzle.

The two DCs have similar measuring ranges, as documented in the manufacturer specifications, with a maximum rainfall intensity value equal to 200 mm h^−1^. The drop water volume, calibrated by the manufacturer, is assumed to be constant and equal to 0.0625 and 0.0600 cm^3^ for the OSC and CRAL, respectively. The area of the collector, measuring range, calibrated drop volume and sensitivity of each instrument are summarized in Table 1.

The sensitivity of the two gauges is equal to 0.005 mm and 0.004 mm for the OSC and CRAL, respectively, which is two orders of magnitude lower if compared with traditional tipping-bucket rain gauges. This characteristic makes this kind of instrument particularly suitable to measure very low rainfall occurrences.

The time resolution of the two instruments is different: the OSC provides the number of drops counted every 10 s, while the CRAL was set to count the drops falling over a one-minute time interval.

### 2.2. Laboratory Test

The laboratory tests were performed at the WMO/CIMO Lead Centre “B. Castelli” on Precipitation Intensity, according to the recommended calibration procedures developed during the WMO Laboratory Intercomparison of Rainfall Intensity gauges [6] and described in the European standard EN 17277:2019 [10].

The calibration system was composed by a constant head water tank that feeds two volumetric pumps (Ismatec Reglo-Z digital and Reglo-CPF digital). The two pumps generate a wide range of known and constant flow rates, which are dispensed into the gauge collector to simulate different rainfall intensities. The water measured by the gauge during the test was then collected in a tank and weighed by means of a precision balance (Mettler Toledo PB4002-s, precision 0.1 g) to measure the total amount of water provided by the system. Assuming a constant value of the water density of 1000 kg m^−3^, the equivalent RI reference value was calculated.

A dedicated software was developed to automatically control the flow rate generation and to measure the reference volume of water, whereas the DC rain gauge measurements were acquired using a dedicated data-logger.

Following the European standard EN 17277:2019 [10], all measurements were aggregated at the one-minute time scale, as recommended by the WMO [6], and the two DC gauge models were tested under various rainfall intensities within the measuring range of each instrument. For the purposes of the present study, the number of RI values tested for each gauge was larger than suggested by the European standard; in particular, for the CRAL gauge, we further investigated the range of low RI values (from 2 to 15 mm h^−1^).

The instrument performance was evaluated using the percentage relative error (e_rel_), defined as:

(1) $$e_{\mathrm{rel}}=\frac{{\mathrm{RI}}_{\mathrm{meas}}-{\;\mathrm{RI}}_{\mathrm{ref}}}{{\mathrm{RI}}_{\mathrm{ref}}}\times 100$$

where the measured one-minute rainfall intensity (RI_meas_) is calculated by adopting the constant drop volumes provided by the manufacturers of each instrument, while the reference rainfall intensity (RI_ref_) is provided by the calibration system. Calibration results for each test are then reported in the form of a non-parametric representation using boxplots, where the lower and upper dots represent the 5th and 95th percentiles, 80% of the data are contained within the two whiskers (10th and 90th percentiles), and half of the data are enclosed within the grey box. The horizontal thin and bold lines indicate the median and the mean of each sample, respectively.

### 2.3. Field Data Analysis

Two co-located OSC rain gauges (PG-51001 and PG-50002 in Table 2) recorded precipitation data at the HKIA field test site from 2012 to 2013. During these two years of observations, the two DC gauges were calibrated, at least once a year, in the HKIA laboratory and the drop volumes dispensed by the nozzle of each gauge at various calibration dates are reported in Table 2. These drop volumes were adopted to calculate the RI values measured by the two instruments, under a constant drop volume assumption, for 55 precipitation events.

Field data for the CRAL gauge are available from the CFARR [25], where a DC gauge is installed since 2001. RI data were calculated for 516 precipitation events selected in the period from 2001 to 2020 starting from the number of drops detected by the gauge at the resolution of 1 min, using the constant drop volumes provided by manufacturer and reported in Table 1.

The calibration curves obtained from the laboratory tests were adopted to calculate the corrected RI values (RI_corr_) for each event measured by the three investigated DC gauges starting from the number of drops measured every minute. A variable drop volume was adopted, calculated as a function of the drop frequency recorded by the gauge.

With the objective to quantify the effect induced by the constant drop volume assumption, the RI relative difference (RI_diff_) between the two approaches was calculated as follow:

(2) $${\mathrm{RI}}_{\mathrm{diff}}=\frac{{\mathrm{RI}}_{\mathrm{meas}}-{\;\mathrm{RI}}_{\mathrm{corr}}}{{\mathrm{RI}}_{\mathrm{corr}}}\times 100$$

## 3. Results

### 3.1. Laboratory Calibration

Since the DC rain gauges count the water drops generated by the dispensing nozzle, the measured variable is the frequency of falling drops and the RI is derived by assuming that the drop volume is known. However, due to the physical processes involved in the formation and detachment of drops from the nozzle inside the gauge, a one-to-one relationship between the drop frequency and the RI only holds beneath a certain RI value. This limit is due to the difficulties of the instrument to generate a series of individual and separated drops when increasing RI. Rather, alternated continuous water trickles and periods of no flow occur, that are interpreted by the instrument as indicative of a lower RI, with a high attribution uncertainty.

**Figure 2:**
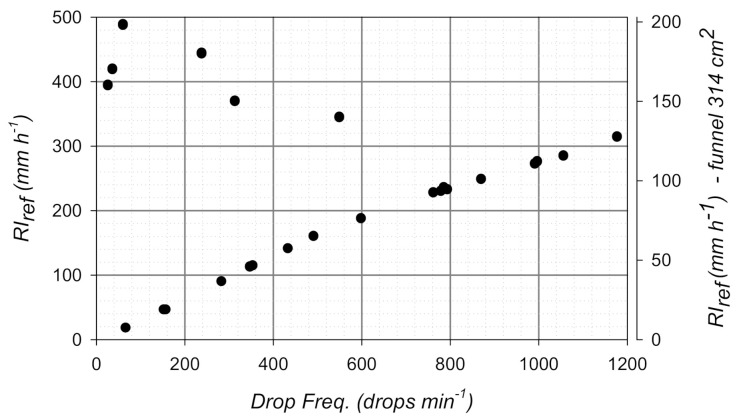
Example of the relationship between the measured drop frequency and RI_ref_. Results refer to the OSC gauge with a funnel area of 127 cm^2^ (left-hand axis) and 314 cm^2^ (right-hand axis).

A dedicated set of calibration tests was conducted to evaluate the relationship between the reference RI and the drop frequency measured by the gauge. As an example, results for the OSC gauge are plotted in Figure 2. Note that the frequency of the dispensed drops continuously increases up to a maximum of about 300 mm h^−1^ (125 mm h^−1^ for a gauge with a funnel area of 314 cm^2^), beyond which it abruptly decreases and the relationship between RI_ref_ and the drop frequency becomes undefined. This indicates that the generated drop volume is not constant, and the mean volume of the drops is significantly larger than the declared one. Beyond 300 mm h^−1^, the performance of the DC gauge abruptly changes, indicating that the operational limit of the rain gauge is reached (see Figure 2).

This behavior, with a given drop frequency being associated with a pair of RI values, undermines the overall reliability of the instrument, even in the low RI range. Moreover, it is impossible to provide a method for the correction of the instrument beyond that limit, to make it compliant with the WMO specifications. Since in the field the reference (actual) RI is not known apriori, the use of a co-located traditional rain gauge (e.g., a tipping-bucket or a weighing gauge) is mandatory for any operational application of this type of gauge.

**Figure 3:**
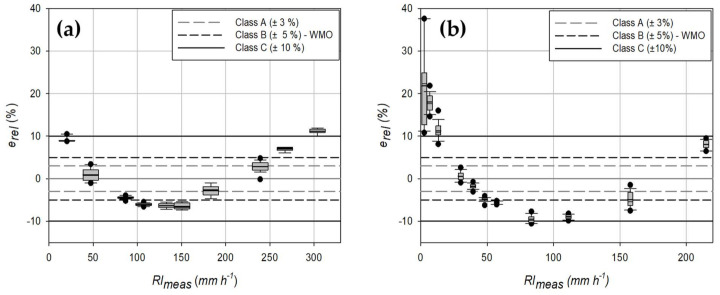
Dynamic calibration results for (**a**) the OSC and (**b**) the CRAL gauges, assuming a constant volume for the generated drops. Horizontal lines indicate the limit of the European standard classification and the WMO recommendation.

Dynamic calibration results (Figure 3a), assuming a constant volume for the generated drops, show that the OSC gauge does not fulfill the WMO recommendation (corresponding to class B in the classification provided by the European standard, with |*e_rel_*|≤ 5%) for most of the operational range. An even worst result is obtained for the CRAL gauge (Figure 3b), which shows a percentage relative error *e_rel_* larger than 10% (class C of the European standard) for RI lower than 10 mm h^−1^. Both gauges overestimate precipitation for low RI values, with positive relative errors, then *e_rel_* decreases with increasing RI, until reaching a minimum underestimation value (*e_rel_* < 0). Beyond this, the error starts growing again until the instrument operational limit is reached, where both gauges overestimate the actual precipitation (*e_rel_* > 0).

The width of the boxplots reported in Figure 3 indicates the precision of the measurement. Both instruments exhibit a high precision at all the tested RI values, except for the CRAL rain gauge at low RI (about 2 mm h^−1^). In this case, due to the very low RI value and the small number of generated drops, the sampling error contributes to the variability of the measurement and therefore degrades the resulting precision.

**Figure 4:**
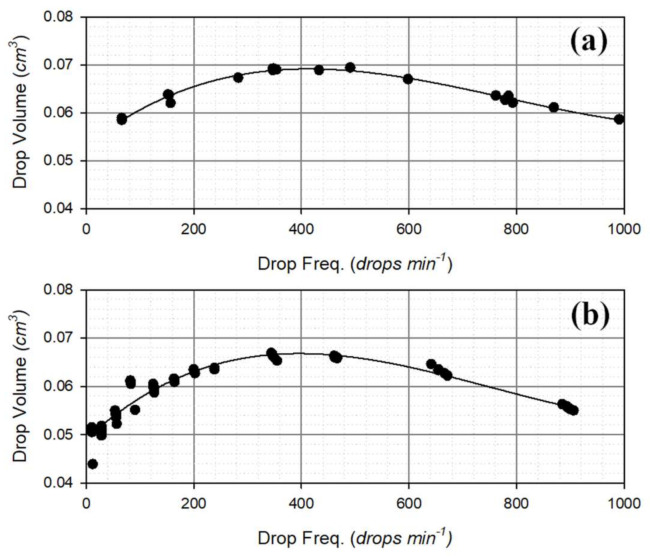
Calibration curves (black lines) and experimental data (black dots) of the generated drop volume as a function of the drop frequency for (**a**) the OSC gauge and (**b**) the CRAL gauge.

Laboratory tests were performed under known and constant flow rates to retrieve the relationship between the drop volume and the drop dispensing frequency measured by the two DC gauges. For each test, the overall water volume was measured by means of a precision balance and the total number of drops, as well as the drop frequency, were recorded. The average drop volume was then calculated by dividing the water volume of each test by the total number of drops recorded by the instrument. Calibration curves (depicted in Figure 4) were obtained by plotting the obtained average drop volume (*DV*) against the measured drop frequency (*DF*) and interpolating the experimental data with a third-order polynomial (with parameters’ values in Table 3):

(3) $$DV=a+b\;\cdot DF+c\;\cdot \;DF^{2}+d\;\cdot DF^{3}$$

Figure 4 shows the variation of the generated drop volume as a function of the drop frequency, therefore the rainfall intensity. Starting from an initial value, valid for very low measured drop frequencies, the drop volume grows with increasing the rainfall intensity until a maximum value is reached approximatively at 400 drops min^−1^ for both instruments. Then, the volume starts decreasing towards the operational limit of the gauge.

The shape of the calibration curves for the two DC gauges is similar, with small differences in the value of the parameters. However, the drops generated by the OSC gauge (Figure 4a) are always larger than the ones generated by the CRAL gauge (Figure 4b), throughout the entire range of drop frequencies. This is ascribable to the internal drop formation mechanism of the gauge, i.e., by the shape and dimension of the nozzle and the rainfall collecting system. For other instruments, larger differences in the shape of the calibration curves could arise in case the mechanism that generates the drops should differ significantly.

**Figure 5:**
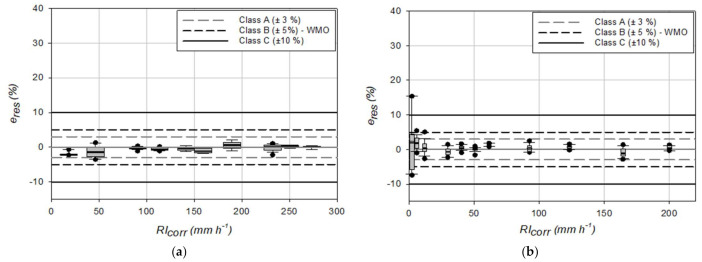
Performance assessment after bias correction according to the calibration curves obtained from dynamic calibration by assuming the actual volume for the generated drops, for (**a**) the OSC gauge and (**b**) the CRAL gauge. Horizontal lines indicate the limit of the European standard classification and the WMO recommendation.

By using the actual volume of the drops, expressed as a function of the measured drop frequency according to the calibration curves, to calculate the RI, the performance of the two instruments falls within the limits of the Class A (|*e_rel_*| < 3%) of the European standard and meet the WMO recommendations for most part of the operational range (Figure 5), except for very low rainfall intensities (less than 2 mm h^−1^).

### 3.2. Field Data Analysis

Results of the field data analysis show that precipitation measurements from DC gauges using a calibrated constant drop volume leads to a general overestimation of the total rainfall amount (RA) of the selected events (Table 4 and Table 5). This is consistent with [19,20], where three dynamically calibrated TBRs were shown to underestimate the precipitation amount when compared to that provided by an OSC gauge using a constant drop volume.

Although this behavior is evident at the two examined field test sites, it appears more prominent at the CFARR site, where a CRAL gauge is employed and the total overestimation of RA ranges between 15.1 and 17.7% (Table 5). At the HKIA site, this value is halved and varies between 7.32 and 8.48 %, with small differences between the two employed OSC gauges (Table 4).

As shown in Figure 4, the actual drop volumes are lower than the nominal (constant) volume in the low and high drop frequencies (and therefore RI) regions, while it is the opposite at intermediate RIs. Therefore, the local climatology and the rainfall variability within typical events play a key role in determining a resulting underestimation or overestimation of the precipitation amount (*RA*) at the event scale.

**Figure 6:**
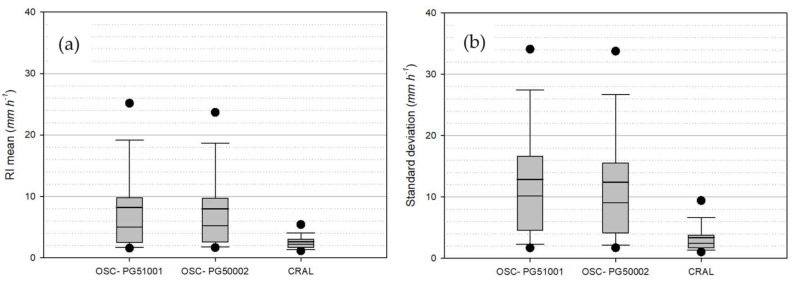
Non-parametric distribution of (**a**) the average value and (**b**) the standard deviation of the one-minute RI calculated for each precipitation event for the two OSC gauges and the CRAL gauge.

In Figure 6, the mean and standard deviation of the one-minute RI values recorded by the DC gauges per each selected precipitation event are shown using the non-parametric boxplot representation. Precipitation events at the HKIA test site (measured by the OSC gauges) show larger mean values and more spread distribution of the mean RI with respect to the CFARR site, and also larger standard deviation values. This indicates that, due to the local rainfall climatology, the two OSC gauges at the HKIA site experienced a wide variety of low to high RI values, leading to a lower overestimation of the RA with respect to the overestimation showed by the CRAL gauge at the CFARR site, where the precipitation regime is characterized by lower and more uniform RI values.

**Figure 7:**
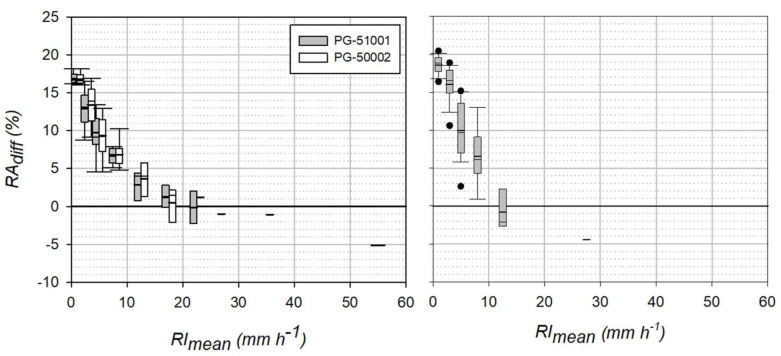
Non-parametric distribution of the relative difference between the total rainfall event amounts (RA_diff_) obtained under the constant and variable drop volume assumption, as a function of RI_mean_ for (**left**) the OSC gauges (at HKIA) and (**right**) the CRAL gauge (at CFARR).

The rainfall events measured at the two field test sites were interpreted using the constant and variable *DV* approaches and classified according to the mean RI obtained using a variable drop volume. Relative RA differences are calculated per each rainfall event as follows:

(4) $${\mathrm{RA}}_{\mathrm{diff}}=\;\frac{{\mathrm{RA}}_{\mathrm{const}}-{\;\mathrm{RA}}_{\mathrm{var}}}{{\mathrm{RA}}_{\mathrm{var}}}\;\times 100$$

where RA_const_ is the total amount obtained under the constant drop volume assumption and RA_var_ is the total amount obtained by letting the drop volume to vary with the rainfall intensity. The variability of RA_diff_ over the whole set of events analyzed is reported in the form of boxplots in Figure 7 as a function of the mean event rainfall intensity, RI_mean_.

## 4. Discussion

Dynamic calibration analysis of the two DC gauges showed that their performance does not fulfil the WMO recommendations when the constant drop volume calibration suggested by the manufacturers is adopted. Indeed, for some RI values within the declared measurement range, the relative percentage error exceeds 10%, a limit that excludes the gauges even from the low performance class of the recent European standard EN 17277:2019 [10].

The performed laboratory tests showed that the volume of the drops generated by the instrument varies as a function of the drop frequency, and therefore of the measured RI. For both the investigated DC gauge models, a relationship between the drop volume and drop frequency was derived. Using a post-processing adjustment algorithm, based on the correction curves obtained from dynamic calibration in the laboratory, the precision of the instruments was improved, and the obtained results are fully compatible with the WMO required maximum admissible error [2]. Also, the best performing class of the European standard EN 17277:2019 [10] was achieved for most of the measuring range (Class A, with *e_rel_* < 3%). However, the instrument performance in the field may be lower than those observed in the laboratory, due to errors induced by the atmospheric conditions, installation, status of maintenance, etc.

When the overall bias of the total rainfall accumulation was investigated in the field, the three gauges exhibited a similar behavior upon varying the one-minute mean rainfall intensity (RI_mean_). The largest overestimation is observed at the lowest RI_mean_ classes, then overestimation decreases as the RI_mean_ increases and underestimation occurs at high RI_mean_. This reflects the fact that overestimation is evident in the calibration curve at the low to medium one-minute RI values, which typically characterize most part of any rainfall event, while high RI showers have a relatively short duration.

The local climatology, therefore, plays a dominant role, since the gauges at the HKIA site (Figure 6a) are subject to precipitation events characterized by higher values of RI_mean_ than those at the CFARR site (Figure 6b). Limited overall overestimation is therefore observed for all rainfall events (Table 4) with respect to the values obtained at the CFARR site. These are characterized by lower RI at the one-minute resolution, and therefore a larger overestimation at the event scale (Table 5).

Due to the internal drop generation mechanism, laboratory tests also revealed the operational limit of these instruments, given by the RI threshold at which the water flux from the internal nozzle starts to be irregular and stepwise continuous. This leads to the failing of a one-to-one relationship between the measured drop frequency and RI. For this reason, although these instruments are very useful to measure rainfall at low RI, a stand-alone installation is discouraged and a co-located rain gauge, using a different measuring principle, is required to avoid large underestimation of severe rainfall events.

## Figures and Tables

**Table 1:** Main characteristics of the two DC rain gauges.

Rain Gauge		OSC	CRAL
Collector Area	(cm^2^)	127 *	150
Measuring range	(mm h^−1^)	0.25–200	0–200
Drop Volume	(cm^3^)	0.0625 **	0.0600
Sensitivity	(mm)	0.005 **	0.004

* The OSC funnel area is 314 cm^2^ but a funnel reduction system is employed at the HKIA field test site. ** Average value provided by the manufacturer.

**Table 2:** Calibration date and associated drop volume for the two OSC gauges installed at the HKIA field test site.

Serial Number	Date of Calibration	Drop Volume (cm^3^)
PG-51001	2011-05-25	0.0644
	2012-05-24	0.0640
	2012-10-26	0.0634
	2013-10-31	0.0660
PG-50002	2011-07-27	0.0631
	2012-07-31	0.0616
	2013-07-31	0.0654

**Table 3:** Parameters of the calibration curves for the two DC gauges.

Instrument	Parameter			
	*a*	*b*	*c*	*d*
OSC	0.0532	8.76 × 10^−4^	-1.44 ×10^−7^	6.18 ×10^−11^
CRAL	0.0491	1.0 ×10^−4^	1.74 ×10^−7^	7.72 ×10^−11^

**Table 4:** Rainfall amount for the selected events recorded by the two OSC gauges at the HKIA field test site using a constant and a variable drop volume (*DV*).

Year	Gauge	% Recorded Minutes	RA at Constant *DV* (mm)	RA at Variable *DV* (mm)	Percentage Difference (%)
2012	PG-51001	88.3	1401.6	1293.4	8.36
	PG-50002	99.2	1665.8	1535.5	8.48
2013	PG-51001	100.0	2231.1	2079.0	7.32
	PG-50002	97.5	2086.7	1938.0	7.67

**Table 5:** Rainfall amount for the selected events recorded by the CRAL gauge at the CFARR field test site using a constant and a variable drop volume (*DV*).

Year	% Recorded Minutes	RA at Constant *DV* (mm)	RA at Variable *DV* (mm)	Percentage Difference (%)
2001	97.2	1087.9	945.1	15.1
2002	98.2	1304.9	1117.6	16.8
2003	94.8	838.8	716.1	17.1
2004	94.1	824.5	715.8	15.2
2005	99.9	716.9	622.4	15.2
2006	99.6	892.6	779.5	14.5
2007	99.1	988.4	849.8	16.3
2008	96.9	1063.1	914.2	16.3
2009	100.0	941.6	809.4	16.3
2010	94.6	690.5	588.6	17.3
2011	92.3	682.1	585.3	16.5
2012	98.6	1230.4	1057.3	16.4
2013	99.4	934.8	794.2	17.7
2014	91.2	1336.2	1148.6	16.3
2015	99.5	825.9	704.9	17.2
2016	98.4	883.0	764.5	15.5
2017	99.9	860.9	733.0	17.4
2018	97.5	830.9	706.6	17.6
2019	97.4	1057.9	907.5	16.6
2020 *	22.5	372.6	316.6	17.7

* Recorded period from January to March 2020.

## Data Availability

Summary statistics of the selected rainfall events recorded by the Drop Counter gauges used in this work are available online at: http://www.precipitation-intensity.it/Sensors2021_Supplemental_Material.html.
